# High masticatory ability attenuates psychosocial stress: A cross-sectional study

**DOI:** 10.1371/journal.pone.0279891

**Published:** 2023-01-18

**Authors:** Ayako Hashimoto, Aya Nozaki, Hiroko Inoue, Toshiko Kuwano

**Affiliations:** 1 Department of Food and Nutrition, Faculty of Home Economics, Kyoto Women’s University, Kyoto, Japan; 2 Graduate School of Integrated Pharmaceutical and Nutritional Sciences, University of Shizuoka, Shizuoka, Japan; 3 Department of Nutrition and Health Sciences, Faculty of Food and Nutritional Sciences, Toyo University, Gunma, Japan; 4 School of Food and Nutritional Sciences, University of Shizuoka, Shizuoka, Japan; University of Concepcion Faculty of Medicine: Universidad de Concepcion Facultad de Medicina, CHILE

## Abstract

Mastication interventions have previously been shown to alleviate acute stress. However, the relationship between masticatory performance and stress response among individuals remains unclear. This study aimed to examine the relationship between masticatory ability and stress response in young women by measuring the autonomic nerve function and salivary α-amylase activity during psychosocial stress. Eighty women (aged 20.0 ± 1.9 years) were divided into either a low or high masticatory performance group, and the Trier Social Stress Test was conducted. Moreover, the autonomic function was measured at rest, immediately before stress, immediately after stress, and 10 min after stress. The salivary α-amylase activity was also measured at rest, 5 min after stress, and 15 min after stress. The visual analog scale (VAS) was used for subjective stress evaluation. There was a significant increase in the autonomic balance of both groups immediately before stress loading, but whilst the high masticatory ability group showed a return to resting-state levels after stress loading, the low masticatory ability group showed elevated levels after stress loading. Salivary α-amylase activity significantly increased 5 min after stress loading in the low, but not high, masticatory ability group. Furthermore, the VAS scores for tension and confusion after stress were significantly higher in the low masticatory ability group than in the high masticatory ability group. Our findings suggest that high masticatory performance may contribute to alleviating psychosocial stress. This is the first study to clarify the relationship between habitual masticatory performance and psychosocial stress suppression in young women.

## Introduction

Stress is a physiological and psychological response to environmental changes and noxious stimuli that profoundly affect well-being, mood, behavior, and health. The biological response to stress activates the endocrine response of the hypothalamic-pituitary-adrenal (HPA) system and autonomic nervous system response in the hypothalamic-sympathetic-adrenal-medullary (SAM) system [1]. Stress activates the neuroendocrine system via the HPA system, which causes the release of glucocorticoids, including cortisol, which in turn affects various biological functions, such as blood pressure, blood glucose, cardiac contractility, cardiac output, and the immune system [2–4]. Furthermore, when the SAM system is activated, catecholamines are released, resulting in increased blood pressure, sweating, blood glucose levels, and arousal. The stress effects on diseases are supported by experimental findings of animal and human studies showing that the HPA and SAM systems are involved and that various stress stimuli activate these systems.

Stress-related illnesses are prevalent worldwide. Stress and coping behaviors have been associated with the development of diseases, including cardiovascular conditions, cancer, breast cancer, colds, and sleep disorders, as well as worsening of symptoms of various disorders, including asthma, irritable bowel syndrome, ulcerative colitis, arthritis, respiratory diseases, skin disorders, and diabetes [2, 5]. Furthermore, stress is associated with symptoms, including headaches, musculoskeletal pain, gastrointestinal upset, hyperventilation, insomnia, fatigue, and depression.

Therefore, methods for attenuating stress in daily life should be established to maintain health. Chewing is considered an effective stress coping behavior [2, 6–9]. Mastication affects the activity of the HPA system and autonomic nervous system [2]. Chewing under stress conditions has been suggested to attenuate stress-induced increases in plasma corticosterone and catecholamines [10], and stress-induced changes to the hippocampus and hypothalamus [2]. In rodents, chewing on wooden sticks has been found to increase the expression of carbohydrate corticoid receptors induced by restraint stress and to act as a stress reliever [11].

Chewing under stressful conditions has been found to inhibit stress-induced activation of the autonomic nervous system and localized release of catecholamines at sympathetic nerve endings [12–14]. Furthermore, aggressive biting during stress exposure significantly attenuates the stress-induced increase in dopamine metabolism in hypothalamic and limbic regions, and the noradrenaline metabolic turnover [2, 12, 14]. Numerous human studies have reported the stress-reducing effects of chewing [15–18].

Habitual gum chewing has been found to relieve anxiety and mental stress [16, 19–21]. Gum chewing during stress loading is associated with state anxiety and decreased salivary cortisol levels [18]; additionally, chewing and light teeth clenching after stress loading rapidly decreases salivary cortisol levels [22]. Notably, a fast masticatory speed and strong masticatory force cause a greater reduction in mental stress than a slow or weak masticatory force [23, 24]. Based on the analysis of salivary stress markers, Tasaka et al. reported that chewing time affects the endocrine response to mental stress and that continuous chewing for > 10 min effectively reduces stress [23].

As mentioned above, numerous studies have evaluated the change in stress index caused by chewing behavior during and after stress. However, none have previously examined the effect of individual chewing ability on stress response and mental states during acute psychological stress. This study aimed to examine the relationships between masticatory performance and stress response in young Japanese women using the Trier Social Stress Test (TSST) [25], which has been widely used to evaluate psychosocial stress, as well as measures of autonomic nerve function and the noninvasively measurable stress index salivary α-amylase activity, which reflects sympathetic nerve activity [26, 27] and is used as a sensitive marker in psychosocial stress tests [27–30].

## Materials and methods

### Study participants

We used advertisements to recruit 105 women to participate in this cross-sectional study at the University of Shizuoka between October 2016 and October 2017. Based on their effects on autonomic nervous function [31], the exclusion criteria included smoking habits; persistent chronic diseases, including diabetes, hypertension, and heart disease; and medication use. Among the 105 women, 25 were excluded based on the exclusion criteria or due to them dropping out of the study. Finally, we included 80 women aged 18–27 years (mean: 20.4 ± 1.9 years). Before study commencement, the purpose and protocol of this study were explained to all participants; furthermore, all participants provided written informed consent before enrollment. This study was approved by the ethical committee of the University of Shizuoka (approval no. 28–7). Additionally, this study was registered in the UMIN Clinical Trials Registry (UMIN000033104). The protocols were conducted in accordance with the principles of the Declaration of Helsinki and its subsequent amendments.

### Measurement of masticatory performance

Masticatory performance was assessed using color-changeable chewing gum (Masticatory Performance Evaluating Gum XYLITOL, Lotte, Tokyo, Japan) [32–34]. All participants were instructed to rinse their oral cavities with tap water for 15 s before chewing the gum. The participants were instructed to normally chew the gum 60 times. Subsequently, the chewed gum was compressed between two glass plates to a thickness of 1.5 mm in polyethylene films. The mixing ability was measured using a colorimeter (CR-20; Konica-Minolta Sensing, Tokyo, Japan) positioned at the following five points: center and approximately 3 mm above, below, as well as to the right and left of the center of the films using the CIELAB color system’s L*, a*, and b* values. ΔE was calculated using the mean values of the L*, a*, and b* values based on the following equation, where the measured pre-chewing L*, a*, and b* values were 72.3, -14.9, and 33.0, respectively [33, 34]

$$\Delta E=\sqrt{[{\left(L^{*}-72.3\right)}^{2}}+{\left(a^{*}+14.9\right)}^{2}+{\left(b^{*}-33.0\right)}^{2}]$$

ΔE indicates the color changes before and after chewing and can be used to evaluate masticatory performance.

### Psychosocial stress test

**Fig 1:**
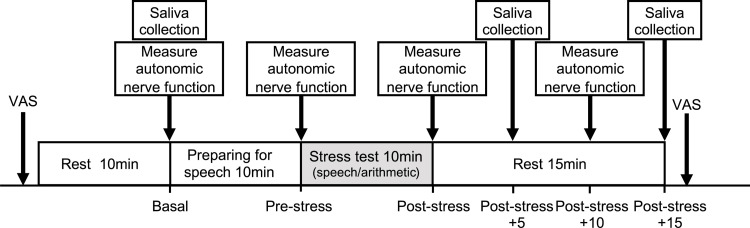
Experimental timelines.

The TSST has been reported to induce profound cardiovascular and endocrine responses in 70–80% of participants [25]. The TSST was performed between 14:40 and 18:10 in a quiet room with air conditioning (room temperature: 24.20 ± 0.76°C). The participants were asked not to exercise, take medication, or consume caffeine after 21:00 on the day before the test and not to eat or drink anything other than water for 2 h before the test. Patients were asked to refrain from excessive exercise on the test day and get sufficient sleep. To eliminate tightness in the body, participants were asked to remove their underwear and change into an inspection gown. After arrival at the laboratory, the participants rested for 10 min after subjective mood evaluation using the Visual Analog Scale (VAS). Next, the participants received explanations regarding two stress tasks in the TSST: a 5-min speech task about personal characteristics and a 5-min mental arithmetic task. They were allowed 10 min to prepare their talks and then undertook the test. Subsequently, the participants rested for 15 min and underwent subjective mood evaluation using the VAS. Autonomic function was measured at four time points: at rest, immediately before the test, immediately after the test, and 10 min after the test. Saliva samples were collected three times: at rest, 5 min after the test, and 15 min after the test (Fig 1).

Time points for subjective mood assessment, autonomic function measurement, and saliva collection.

### Measurement of autonomic nerve activity

Autonomic nerve activity was measured using frequency-domain analyses of the a-a wave intervals of accelerated plethysmography using ARTETT (U-MEDICA, Inc. Co., Osaka, Japan) [35–37]. The a-a intervals were analyzed using the maximum entropy method in the frequency analysis. Low-frequency power (LF%) and high-frequency power (HF%) were calculated as the power within the frequency ranges of 0.04 Hz to 0.15 Hz and 0.15 Hz to 0.4 Hz, respectively. The HF is mediated by the parasympathetic nervous system [38, 39], while the LF originates from various sympathetic and vagal mechanisms [38, 40]. The ratio of LF to HF power (LF/HF) reflects the balance of autonomic nervous activities [41].

### Measurement of α-amylase activity

Saliva samples were collected using salivette (Sarsted, Rommelsdorf, Germany) collection devices and stored on ice until centrifugation at 3,000 rpm for 5 min. The resulting samples were stored at -80°C until analysis, and salivary α-amylase activity was measured using the clinically applied 2-chloro-4-nitrophenyl-4- galactopyranosyl-maltoside (Gal-G2-CNP) method (SRL Co., Tokyo, Japan).

### Assessment of subjective mood

Subjective mood, tension, depression, anger, liveliness, fatigue, and confusion were evaluated using the VAS [42], which comprised 100-mm lines with 0 mm and 100 mm indicating “not at all” and “strongest,” respectively.

### Other variables

The following items were assessed within two weeks before and after the TSST. We measured height, weight, body fat percentage, fat mass, lean mass, body mass index (BMI), and body mass index using the Body Fat Analyzer (Model No. TBF-215, TANITA). Systolic blood pressure, diastolic blood pressure, and heart rate were measured using a digital automatic blood pressure monitor (HEM-5001, OMRON). The average of two consecutive measurements was used for analysis. Grip strength was measured using a digital grip strength meter; GRIP-D (T.K.K. 5101, Takei Kiki Kogyo). Bite force was measured using a dental occlusal force meter; OCCLUSAL FORCE-METER (GM10, Nagano Keiki Co., Ltd.).

### Statistical analyses

The sample size was calculated using G*Power 3 [43]. To achieve an interaction effect size of 0.65 with 80% power at a 0.05 significance level for the *t*-test, the required sample size was 78 participants. To account for a potential 20% attrition rate, we sought to enroll 98 participants.

Based on ΔE, the participants were classified into the following two groups: 1) low masticatory performance (ΔE < 75^th^ percentile, *n* = 59) and 2) high masticatory performance (ΔE ≥ 75^th^ percentile, *n* = 21). Statistical analyses were performed using IBM SPSS statistics version 25 (SPSS, Inc., Chicago, IL, USA). All data were checked for normality and homogeneity using the Shapiro–Wilk W-test before each analysis.

Data regarding autonomic function and salivary α-amylase were skewed; therefore, we performed nonparametric statistics to compare stress indicators at different time points during the TSST. Specifically, we performed a two-way analysis of ranks (Friedman test) followed by Wilcoxon tests for individual comparisons, while Bonferroni’s correction was applied for multiple comparisons.

Depending on the data normality, between-group comparisons were performed using unpaired *t*-tests or Mann–Whitney *U* tests. Paired *t*-tests or Wilcoxon’s signed-rank t were used to compare VAS parameters before and after the TSST. All data are presented as mean ± standard deviation (SD). The significance threshold was set at *p* < 0.05.

## Results

### Characteristics of the participants

There were no significant between-group differences in age, height, body fat percentage, BMI, blood pressure, heart rate, or grip strength. The maximum bite force was significantly higher in the high masticatory ability group than in the low masticatory group (Table 1). Furthermore, there were no significant between-group differences in the depression and stress states assessed using the Self-Rating Depression Scale and Japanese Perceived Stress Scale.

**Table 1:** Basic characteristics of the subjects.

	Low masticatory group (*n* = 59)			High masticatory group (*n* = 21)			*p*			Total (*n* = 80)		
Age (years)	20.4	±	1.9	20.3	±	1.8	0.871		^Ψ^	20.4	±	1.8
Height (cm)	158.5	±	6.4	160.2	±	5.5	0.259		^Φ^	158.9	±	6.2
Weight (kg)	49.7	±	7.5	53.4	±	5.7	0.045	*	^Φ^	50.7	±	7.2
BFP (%)	24.0	±	4.6	26.0	±	4.2	0.149		^Ψ^	24.5	±	4.6
BMI (kg/m^2^)	19.7	±	2.1	20.8	±	2.1	0.063		^Ψ^	20.0	±	2.1
SBP (mmHg)	108.2	±	11.7	107.4	±	6.6	0.961		^Ψ^	108.0	±	10.6
DBP (mmHg)	68.1	±	8.8	68.9	±	6.3	0.707		^Φ^	68.3	±	8.2
Heart rate (bpm)	65.5	±	11.4	62.3	±	13.3	0.082		^Ψ^	64.7	±	11.9
Grip strength (kg)	25.3	±	3.7	26.7	±	4.3	0.171		^Ψ^	25.7	±	3.9
Maximum bite force (kg)	42.1	±	15.4	57.3	±	16.7	0.002	**	^Φ^	46.1	±	17.0
Values of L*, a*, b*, and *ΔE* of masticatory performance-evaluating gum												
L*	59.2	±	1.7	55.6	±	1.7	<0.001	** *	^Φ^	58.3	±	2.3
a*	18.3	±	4.0	26.5	±	2.3	<0.001	** *	^Ψ^	20.5	±	5.1
b*	13.4	±	3.1	7.7	±	2.1	<0.001	** *	^Ψ^	11.9	±	3.8
ΔE	40.8	±	5.1	51.3	±	3.0	<0.001	** *	^Ψ^	43.5	±	6.6
SDS	41.4	±	7.1	41.5	±	6.0	0.984		^Φ^	41.5	±	6.8
JPSS	27.9	±	5.5	28.8	±	5.5	0.298		^Ψ^	28.1	±	5.5

Data are expressed as mean ± standard deviation.

^Φ^Independent samples *t*-test

^Ψ^Mann–Whitney *U* test

*p < 0.05

**p < 0.01

***p < 0.001

BMI: body mass index, BFP: body fat percentage, SBP: systolic blood pressure, DBP: diastolic blood pressure, SDS: Self-Rating Depression Scale, JPSS: Japanese Perceived Stress Scale.

### Effect of psychosocial stress on autonomic nervous system function

**Fig 2:**
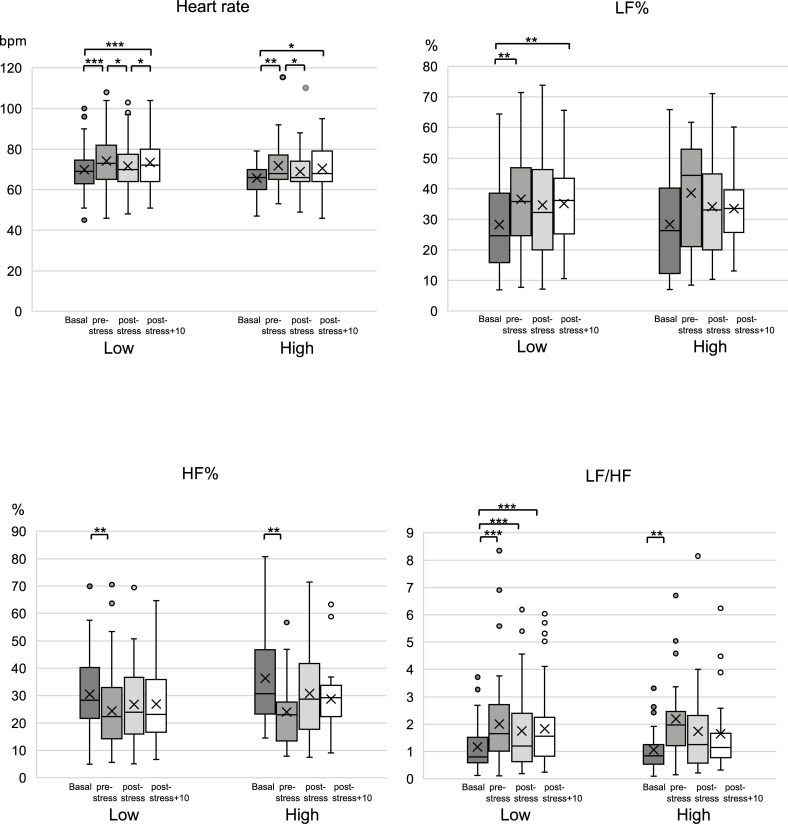
Autonomic nerve activity in response to the TSST. Changes in heart rate (A), LF% (B), HF% (C), and LF/HF (D) in response to the TSST. * indicates significant differences. *p < 0.05, **p < 0.01, ***p < 0.001.

Fig 2 shows the changes in autonomic functions during the TSST. There were no significant between-group differences in pulse rate, LF%, HF%, or LF/HF at rest, immediately before stress, immediately after stress, and 10 min after stress. In both groups, the pulse rate significantly increased from resting state to immediately before the TSST and significantly decreased from immediately before the TSST to immediately after the TSST (low masticatory ability group: *p* < 0.001 and *p* < 0.05, respectively; high masticatory ability group: *p* < 0.01 and *p* < 0.05, respectively).

LF% showed no significant change in the high masticatory ability group; however, in the low masticatory ability group, it was significantly higher immediately before the TSST compared with the resting state (*p* < 0.01). In both groups, HF% significantly decreased immediately before the TSST compared with the resting state (*p* < 0.01). LF/HF, which is an index of autonomic balance, was significantly higher in the high masticatory ability group only immediately before the TSST compared to the resting state (*p* < 0.01). By contrast, the low masticatory ability group showed significantly higher LF/HF values immediately before, immediately after, and 10 min after the TSST compared to the resting state (*p* < 0.001).

### Effect of psychosocial stress on salivary α-amylase activity

**Fig 3:**
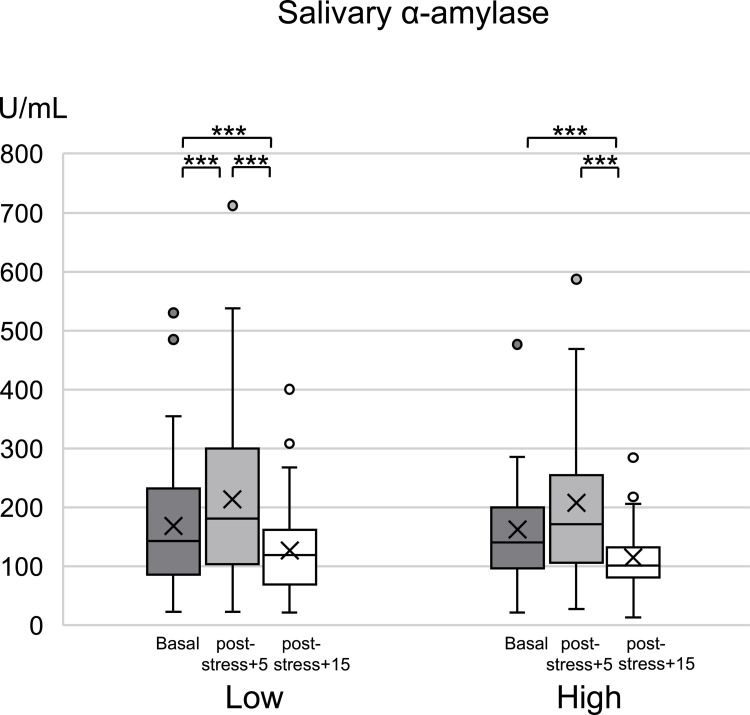
Salivary α-amylase activity in response to the TSST. Changes in salivary α-amylase activity in response to the TSST. * indicates significant differences. ***p < 0.001.

Fig 3 shows the changes in salivary α-amylase activity during the TSST. There was no significant between-group difference in salivary α-amylase activity at rest, 5 min after stress, and 10 min after stress. Only the low masticatory ability group showed a significant increase after 5 min of stress loading compared with the resting state (*p* < 0.001).

### Effect of psychosocial stress on subjective mood

Table 2 shows the VAS results before and after the TSST. In the high masticatory ability group, compared with before the TSST, tension (*p* < 0.01), depression (*p* < 0.05), and confusion (*p* < 0.05) were significantly higher, whereas liveliness (*p* < 0.01) was significantly lower after the TSST. In addition, there was a significant change in all items in the low masticatory ability group. Furthermore, compared with those before the TSST, the scores of tension, depression, anger, fatigue, and confusion were significantly higher (*p* < 0.001), whereas the score of liveliness was significantly lower (*p* < 0.001) after the TSST. Additionally, after the TSST, the low masticatory ability group showed significantly higher scores for tension and confusion than the high masticatory ability group (*p* < 0.05).

**Table 2:** Visual analog scale (VAS) scores.

	Low masticatory group (*n* = 59)						High masticatory group (*n* = 21)						*p*										
													Low masticatory group			High masticatory group			Before stress		After stress		

	Before stress			After stress			Before stress			After stress			Before stress vs. After stress			Before stress vs. After stress			Low masticatory vs. High masticatory		Low masticatory vs. High masticatory		


VAS																							
Tension (mm)	23.2	±	23.0	43.8	±	26.2	15.6	±	15.0	30.4	±	25.4	<0.001	***	^π^	0.003	**	^π^	0.418	^Ψ^	0.041	*	^Ψ^
Depression (mm)	11.1	±	17.1	25.0	±	24.2	13.8	±	22.7	17.2	±	21.0	<0.001	***	^π^	0.030	*	^π^	0.884	^Ψ^	0.204		^Ψ^
Anger (mm)	6.2	±	11.2	14.3	±	18.1	7.5	±	12.5	9.3	±	13.2	<0.001	***	^π^	0.139		^π^	0.950	^Ψ^	0.361		^Ψ^
Liveliness (mm)	35.7	±	22.3	26.1	±	22.1	26.1	±	17.8	20.1	±	16.6	<0.001	***	^π^	0.009	**	^∬^	0.077	^Ψ^	0.325		^Ψ^
Fatigue (mm)	27.5	±	22.8	42.4	±	24.9	26.3	±	23.5	33.2	±	27.0	<0.001	***	^π^	0.221		^π^	0.926	^Ψ^	0.161		^Φ^
Confusion (mm)	12.9	±	19.0	36.7	±	27.7	13.0	±	18.2	24.5	±	28.0	<0.001	***	^π^	0.013	*	^π^	0.772	^Ψ^	0.036	*	^Ψ^

Data are expressed as mean ± standard deviation.

^∬^Paired *t*-test, ^π^Wilcoxon’s signed-rank test

*p < 0.05

**p < 0.01

***p < 0.001.

^Φ^Independent samples *t*-test

^Ψ^Mann–Whitney *U* test: *p < 0.05, **p < 0.01.

## Discussion

This study aimed to clarify the relationship between masticatory ability and stress response in young women by examining autonomic nerve function and salivary α-amylase activity over time during the TSST. Regarding autonomic nerve function, LF/HF remained significantly increased in the low masticatory ability group (rather than the high masticatory ability group) at 10 min after stress loading compared with the resting state. Additionally, unlike the high masticatory ability group, the low masticatory ability group showed a significant increase in salivary α-amylase activity after 5 min of stress loading compared with the resting state. This suggests that masticatory ability may affect the suppression of sympathetic nerve excitation caused by psychological stress. Furthermore, assessment of subjective mood using the VAS after stress loading revealed significantly higher scores for tension and confusion in the low mastication performance group than in the high mastication performance group. These results suggest that increasing masticatory ability may attenuate objective and subjective stress during psychological stress loading. Hence, there is a need for nutritional education regarding enhancing the habitual chewing ability for stress prevention.

The values for LF/HF, which is an index of resting-state autonomic balance, remain to be internationally standardized. Several studies have reported that the resting-state LF/HF values range from 1.3 to 2.2 and 1.5 to 2.0 [44, 45]. However, a study reported that LF/HF at rest was 0.56 (0.39–1.67) (median and interquartile range), which increased to 2.92 (2.44–3.69) after mental fatigue stress loading [37]. Among our participants, the mean LF/HF at rest in the low and high masticatory ability groups was 1.17 and 1.05, respectively, which indicates a very restful state. The respective mean values of LF/HF in the low and high masticatory ability groups were 2.00 and 2.19 immediately before stress, 1.75 and 1.73 immediately after stress, and 1.83 and 1.65 at 10 min after stress. In both groups, stress was induced immediately before stress loading. The induced high-stress status continued and normalized in the low and high masticatory performance groups, respectively. The observed difference in response to psychological stress depending on the masticatory ability is a novel finding.

In the high masticatory ability group, there was no significant change in LF% during stress loading. However, in the low masticatory ability group, compared with the resting state, there was a significant increase in LF% immediately before stress loading, immediately after stress loading, and after 10 min. Contrastingly, HF% was significantly decreased in both groups compared with the resting state immediately before stress loading; however, there was no significant difference immediately after, and 10 min after stress loading. This suggests that the significant increase in LF/HF immediately before and after stress loading, as well as after 10 min, compared with the resting state in the low masticatory ability group was strongly affected by the increase in LF%. In other words, the habitual masticatory ability may affect the suppression of sympathetic nerve excitation induced by psychological stress. Koizumi et al. used a rat model to demonstrate that mastication can suppress stress-induced increases in LF/HF, attenuate the stress-induced increase in noradrenaline concentration, and attenuate stress-induced arrhythmia [46]. This could be associated with the act of mastication itself, as well as the individual’s habitual masticatory ability.

Several studies have reported the stress-relieving effect of chewing. Regarding acute stress, chewing a gum after the Uchida–Kraepelin test decreased the salivary chromogranin A levels, which are stress indicators [15]. After performing arithmetic calculations, gum chewing and clenching were revealed to decrease salivary cortisol levels [22, 24]. When participants completed the TSST while chewing a gum, there was a significant reduction in subjective stress compared with those who did not chew gum [47]. Gum chewing during multitasking stress load was associated with reduced anxiety, stress, and salivary cortisol levels [18].

Regarding habitual chewing, individuals with a habit of gum chewing present with less chronic stress [19, 20]. Furthermore, a 2-week intervention trial on gum chewing in college students reported that it reduced stress, anxiety, and depression [16]. These reports clearly demonstrate the effects of daily gum chewing [17].

The recovery of LF/HF to resting-state values immediately after stress loading and after 10 min in the high masticatory ability group suggests that daily masticatory ability may affect and contribute to the suppression of sympathetic nerve excitation caused by psychological stress. Stress reduction may be facilitated by increasing the frequency of habitual chewing and improving masticatory ability.

During stress conditions, α-amylase is secreted through the direct action of the sympathetic nervous system and SAM system activation [1, 48]. Compared with salivary cortisol, salivary α-amylase shows a faster response to stress load; moreover, it has been used as an indicator for capturing changes in sympathetic nerve activity and measuring autonomic function [22, 30]. Several studies have shown that salivary α-amylase activity is maximal during or immediately after TSST stress, before rapid decreases [22, 49]. In our study, there was a significant increase in salivary α-amylase activity in the low masticatory ability group at 5 min after stress loading compared with the resting state. However, this significant difference was not observed in the high masticatory ability group. These results further indicate that high masticatory strength may affect the suppression of sympathetic nerve excitation resulting from psychological stress.

In both groups, the VAS scores revealed subjective stress induced by the TSST. However, after stress loading, there were significantly higher scores for tension and confusion in the low masticatory ability group than in the high masticatory ability group. In summary, participants with higher masticatory ability are less likely to feel subjective stress.

A strength of this study is that we could compare the psychological stress responses of young women with different habitual masticatory performances using objective and subjective indices under uniform conditions, including test time, mealtime, room temperature, clothing, and caffeine restriction, which may affect the autonomic nervous system function. However, this study is not without limitations. First, the study included only Japanese women within a narrow age range; therefore, it is necessary to consider whether the results of this study can be generalized. Second, since this was a cross-sectional study, the causal relationship between chewing ability and stress reduction remains unclear. Third, we only used autonomic nerve function, salivary α-amylase, and subjective evaluation as stress indicators; the effect on the HPA pathway should also be investigated. Therefore, future longitudinal and intervention studies on mastication should examine the effect of mastication ability on stress reduction in more detail.

## Conclusions

This study provides novel findings regarding changes in autonomic nerve function and salivary α-amylase activity in psychosocial stress resulting from mastication ability. Our results suggest that habitual mastication ability may suppress sympathetic nerve excitation and relieve stress. This study provides further evidence for mastication as an alternative for stress mitigation.

## Supporting information

S1 Table Minimal underlying data set. (XLSX)

S2 Table Autonomic nerve activity in response to the TSST. (PDF)

S3 Table Salivary α-amylase activity in response to the TSST. (PDF)
