# Supplementary material for: High masticatory ability attenuates psychosocial stress: A cross-sectional study
Source: PLoS One. 2023 Jan 18;18(1):e0279891. doi: 10.1371/journal.pone.0279891 (PMC9847911; doi:10.1371/journal.pone.0279891)
Supplement: S2 Table — (PDF) [file pone.0279891.s002.pdf]

S2 Table. Autonomic nerve activity in response to the TSST.

		Basal	pre-stress	post-stress	post-stress +10 min	*p*
Heart rate (bpm)	Low masticatory group (n=59)	69.9 ± 10.7^{a}	74.1 ± 12.6^{b}	71.7 ± 11.9^{ac}	73.4 ± 11.2^{b}	<0.001 ***
	High masticatory group (n=21)	65.7 ± 8.0^{a}	71.8 ± 12.5^{b}	69.0 ± 11.4^{ac}	70.5 ± 10.9^{bc}	<0.001 ***
LF% (%)	Low masticatory group (n=59)	28.2 ± 14.2^{a}	36.5 ± 15.8^{b}	34.7 ± 16.6^{ab}	35.2 ± 14.0^{b}	0.031 *
	High masticatory group (n=21)	28.4 ± 16.3	38.6 ± 16.8	34.0 ± 17.3	33.5 ± 12.6	0.113
HF% (%)	Low masticatory group (n=59)	30.5 ± 13.4^{a}	24.5 ± 13.5^{b}	26.8 ± 13.1^{ab}	27.0 ± 14.4^{ab}	0.046 *
	High masticatory group (n=21)	36.4 ± 18.7^{a}	24.0 ± 12.7^{b}	30.7 ± 18.0^{ab}	28.8 ± 13.6^{ab}	0.027 *
LF/HF	Low masticatory group (n=59)	1.17 ± 0.86^{a}	2.00 ± 1.51^{b}	1.75 ± 1.39^{b}	1.83 ± 1.40^{b}	<0.001 ***
	High masticatory group (n=21)	1.05 ± 0.87^{a}	2.19 ± 1.63^{b}	1.73 ± 1.80^{ab}	1.65 ± 1.50^{ab}	0.002 *

Data are expressed as mean ± standard deviation.

Two-way analysis of ranks (Friedman test): $*p < 0.05$, $**p < 0.01$, $***p < 0.001$

Different letters indicate significant differences ($p < 0.05$) according to the Wilcoxon tests with a Bonferroni's correction for multiple comparisons.
