# Supplementary material for: High masticatory ability attenuates psychosocial stress: A cross-sectional study
Source: PLoS One. 2023 Jan 18;18(1):e0279891. doi: 10.1371/journal.pone.0279891 (PMC9847911; doi:10.1371/journal.pone.0279891)
Supplement: S3 Table — (PDF) [file pone.0279891.s003.pdf]

S3 Table. Salivary α-amylase activity in response to the TSST.

		Basal	post-stress +5 min	post-stress +15 min	p
α-amylase activity (U/mL)	Low masticatory group (n=59)	168.0 ± 105.0[a]	214.0 ± 140.6[b]	126.9 ± 75.8[c]	<0.001 ***
	High masticatory group (n=21)	162.8 ± 102.5[a]	207.9 ± 146.0[a]	115.0 ± 67.4[b]	<0.001 ***

Data are expressed as mean ± standard deviation.

Two-way analysis of ranks (Friedman test): ***$p < 0.001$

Different letters indicate significant differences ($p < 0.05$) according to the Wilcoxon tests with a Bonferroni's correction for multiple comparisons.
